# Xenon in ALS Treatment: What Are We Waiting for?

**DOI:** 10.1111/cns.70435

**Published:** 2025-05-10

**Authors:** Ferhat Çelik

**Affiliations:** ^1^ Dicle University Atatürk Vocational School of Health Services Diyarbakir Turkey

Dear Editor,

Glutamate, the most abundant neurotransmitter in the central nervous system (CNS), is the key regulator of neuronal excitability. It is well established that glutamate exerts its effects through three main ionotropic receptors—N‐methyl‐D‐aspartate (NMDA), α‐amino‐3‐hydroxy‐5‐methyl‐4‐isoxazolepropionic acid (AMPA), and kainate receptors—to maintain synaptic balance and drive neurotransmission [1]. NMDA receptors (NMDARs) are particularly notable for their high permeability to Ca^2+^ ions, a property that, when combined with intracellular calcium overload, endoplasmic reticulum stress, and mitochondrial dysfunction, can trigger excitotoxicity mechanisms [2].

Dysfunctions in the glutamatergic system have long been investigated in relation to neurodegenerative diseases, particularly in the context of Amyotrophic Lateral Sclerosis (ALS). Analyses of spinal cord and cortical tissues from ALS patients have revealed significant reductions in the function of glutamate transporter proteins [3]. Similarly, postmortem studies have reported impairments in glutamate metabolism. Moreover, mutations associated with ALS, such as SOD1, C9ORF72, TARDBP, and FUS, have been shown to have a direct connection to dysregulation within the glutamatergic system [4].

Xenon (Xe), which has been used as a safe anesthetic agent since the mid‐20th century, has also garnered attention for its neuroprotective properties over the past two decades. The first study demonstrating Xenon's neuroprotective effects was published in 2003, using rat models of ischemic brain injury [5]. Subsequent research has shown that Xenon preserves neurocognitive functions and reduces neuronal loss in conditions such as focal ischemia and perinatal hypoxic–ischemic injury [6, 7].

One of the key mechanisms underlying Xenon's anesthetic and neuroprotective effects is its antagonistic action on NMDA receptors [8]. NMDA receptor inhibition may serve as a protective mechanism against glutamate excitotoxicity in diseases such as ALS. Xenon's ability to easily cross the blood–brain barrier and rapidly enter and exit brain cells makes it pharmacologically advantageous [9]. In addition to NMDA antagonism, it has also been reported to inhibit AMPA and kainate receptors; however, the significance of these effects in the context of ALS remains unclear [9].

Xenon's potential neuroprotective effects in ALS are not limited to NMDA receptor antagonism. There is evidence suggesting that it may also influence key pathophysiological mechanisms of ALS, including apoptotic cell death, oxidative stress, and microglial dysfunction [10].

Given all these findings, it is evident that Xenon should be investigated as a potential therapeutic agent for ALS. However, despite its promising mechanisms, there are currently no clinical or preclinical studies directly examining the relationship between Xenon and ALS in the literature. We emphasize the need for extensive research, ranging from cell culture studies to animal models, to explore this potential.

We urge the scientific community to conduct in‐depth investigations into Xenon's potential neuroprotective effects in ALS.

## Conflicts of Interest

The author declares no conflicts of interest.

## Data Availability

Data sharing not applicable to this article as no datasets were generated or analysed during the current study.
